# A Tutorial of the Poisson Random Field Model in Population Genetics

**DOI:** 10.1155/2008/257864

**Published:** 2008-07-02

**Authors:** Praveen Sethupathy, Sridhar Hannenhalli

**Affiliations:** ^1^Department of Genetics, School of Medicine, University of Pennsylvania, Philadelphia, PA 19104, USA; ^2^Department of Computer and Information Sciences, School of Engineering and Applied Sciences, University of Pennsylvania, Philadelphia, PA 19104, USA

## Abstract

Population genetics is the study of allele frequency changes driven by various evolutionary forces such as mutation, natural selection, and random genetic drift. Although natural selection is widely recognized as a bona-fide phenomenon, the extent to which it drives evolution continues to remain unclear and controversial. Various qualitative techniques, or so-called “tests of neutrality”, have been introduced to detect signatures of natural selection. A decade and a half ago, Stanley Sawyer and Daniel Hartl provided a mathematical framework, referred to as the Poisson random field (PRF), with which to determine quantitatively the intensity of selection on a particular gene or genomic region. The recent availability of large-scale genetic polymorphism data has sparked widespread interest in genome-wide investigations of natural selection. To that end, the original PRF model is of particular interest for geneticists and evolutionary genomicists. In this article, we will provide a tutorial of the mathematical derivation of the original Sawyer and Hartl PRF model.

## 1. Introduction

Selectionists and neutralists have fiercely debated, for the past five decades, the extent to which Darwinian selection has shaped molecular evolution. However, both camps do agree that Darwinian selection is a bona fide natural phenomenon. Therefore, various so-called “tests of neutrality” have been developed to detect natural selection on a particular gene or genomic location (for a review on this topic, see [1]). However, these tests are often qualitative and only provide the directionality of selection. A decade and a half ago, S. Sawyer and D. Hartl provided a mathematical framework with which to determine quantitatively the intensity of selection on a particular gene, which they applied to the *Adh* locus in the *Drosophila* genome [2]. This framework is referred to as the Poisson random field (PRF) model. They then further used this framework to analyze codon bias in enteric bacteria [3]. Owing to the recent availability of whole genome sequences and genome-wide human polymorphism data, it has become increasingly tractable to perform genome-wide scans for signatures of selection. The PRF model has been applied to estimate the intensity of selection on synonymous and nonsynonymous sites throughout mitochondrial and nuclear genomes of a variety of species, including human [4–12]. Very recently, due to the advent of high-throughput experimental and computational identification of genomic regulatory elements, there has been an interest to estimate the intensity of natural selection on regulatory mutations. Chen and Rajewsky [13] use the PRF, among other techniques, to provide evidence for purifying selection (even stronger than on nonsynonymous coding sites) on a class of regulatory sites known as microRNA target sites. Due to the potentially wide range of applications of, and opportunities for theoretical extensions to, the PRF model, it is an increasingly important mathematical framework for quantitative geneticists. In this article, we will provide a tutorial of the mathematical derivation of the basic PRF model that was originally developed in [2]. The tutorial will follow the outline provided below:

- Wright-Fisher model,
- diffusion approximation to the Wright-Fisher model,
- derivation, via diffusion theory, of formulas describing evolutionary processes of interest,
- derivation of the PRF using the above-mentioned formulas.

The first three items are discussed in [14], and the last point was originally presented in [2]. In this tutorial, we aim to provide an integrated and comprehensive presentation that is accessible to nonprofessionals or beginners in the field of population genetics. Since the primary purpose is to review mathematical derivations, familiarity with calculus and at least a cursory knowledge of genetics will be helpful for the reader.

## 2. The Wright-Fisher Model

The Wright-Fisher (WF) model describes the change in frequency of a single mutation (derived allele) in a population over time. The simplest version of the model makes the following assumptions: (1) nonoverlapping generations, (2) constant population size in each generation, and (3) random mating, and is described as follows.

Consider a population of *N* diploid individuals that has a single polymorphic site with two alleles, one ancestral and one derived. Under this model, the frequency of the derived allele in the current generation is a function of the selection pressure on this allele and the binomial sampling effect with probabilities proportional to the frequency of this allele in the previous generation. The probability, *p_ij_*, that there are *j* genes of the derived allele present at generation G + 1 given *i* genes of the derived allele present at generation G is given by the following binomial calculation:

(1) $$p_{ij}\;=\;\left(\begin{array}{c} 2N\\ j \end{array}\right)\;{\left(\Psi_{i}\right)}^{j}\;{\left(1-\Psi_{i}\right)}^{2N-j},$$

where Ψ_*i*_ depends on the relative fitness of the derived allele.

Assuming no dominance and no recurrent mutation,

(2) $$\Psi_{i}\;=\;\frac{x(1+s)}{x(1+s)+(1-x)},$$

where 1 + *s* is the fitness of the derived allele relative to 1 for the ancestral allele, and *x* (which is simply *i*/2*N*) is the derived allele frequency (daf) in generation G. In the simplest model (no selection and no recurrent mutation), Ψ_*i*_ is simply *x* or *i*/2*N*.

**Figure 1:**
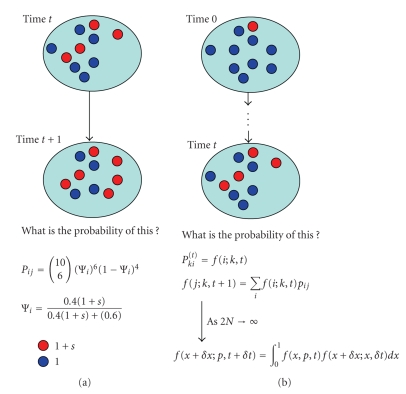
Pictorial representation of the Wright-Fisher process and its diffusion approximation: (a) basic Wright-Fisher model assuming selection, but no dominance or recurrent mutation and (b) diffusion approximation to the basic Wright-Fisher model.

The intuition behind Ψ*_i_* is the following. Consider the scenario where both the ancestral and the derived alleles are neutrally evolving (no or negligible selection pressure). In this case, the probability of sampling a gene of the derived allele from the population in generation G is simply the frequency of the derived allele in generation G, *i*/2*N* or *x*. This can be rewritten as *x*/[*x* + (1 − *x*)]. Now, suppose that the derived allele is under some selection, *s*, meaning that the fitness of the derived allele is 1 + *s* relative to 1 for the ancestral allele. In this case, genes are sampled according to their relative fitnesses (as in the equation for Ψ*_i_* above). Figure 1(a) provides a pictorial representation of the basic Wright-Fisher model.

## 3. Diffusion Theory

We define *p*_*ki*_^(*t*)^ as the probability that a polymorphic site has *i* genes of the derived allele at time *t*, given that it had *k* genes of the derived allele at time 0. *p*_*ki*_^(*t*)^ satisfies the following:

(3) $$p_{kj}^{\left(t+1\right)}=\sum_{i}p_{ki}^{\left(t\right)}\;p_{ij},$$

where *p*_*ij*_ is given in (1).

It is convenient to change notation and write *p*_*ki*_^(*t*)^ as *f*(*x*; *p*, *t*), so that the above becomes

(4) $$f(j;\;k,t+1)=\sum_{i}f(i;\;k,t)\;p_{ij}.$$

In this framework, it has been shown to be extremely difficult to explicitly derive formulas for several quantities of evolutionary interest. However, as the size of the population approaches infinity (i.e., *N* → ∞), and assuming that the scaled selection pressure (*Ns*) and scaled mutation rate (*N*μ**) remain constant, the discrete Markov process given above can be closely approximated by a continuous-time, continuous-space diffusion process (Figure 1(b)):

(5) $$f(x+\delta x;\;p,t+\delta t)=\int_{0}^{1}f(y;\;p,t)f(x+\delta x;\;y,\delta t)\;dy,$$

where *f*(*x*; *p*, *t*) is the probability distribution of *x* at time *t*, *x* is the daf at time *t*, *p* is the daf at time 0, and *δx* is the daf change in time *δt*.

We can perform a Taylor series expansion on both sides in *δt* and *δx* to derive the forward Kolmogorov equation:

(6) $$\frac{\partial f(x;p,t)}{\partial t}\;=\;\frac{\partial^{2}[b(x)f(x;p,t)]}{2\partial x^{2}}\;-\;\frac{\partial [a(x)f(x;p,t)]}{\partial x},\;$$

where

(7) $$\begin{array}{r} E(\Delta x)\approx a(x)\;dt,\\ \text{var}\;(\Delta x)\approx b(x)\;dt, \end{array}$$

and *a(x)* and *b(x)* depend on the genetic model (e.g., see eq (24).

**Figure 2:**
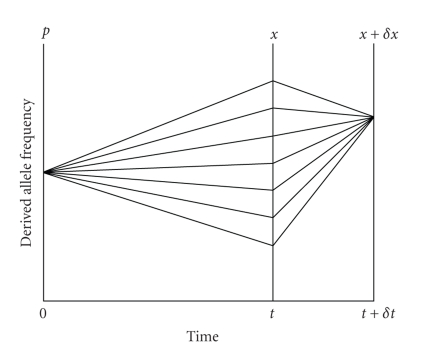
A diagrammatic intuition for (3) illustrates that the probability of derived allele frequency *x* + *δx* at time *t* + *δt* is the product of the probability of moving from *p* to *x* in time *t* and the probability of moving from *x* to *x* + *δx* in time *δt*, summed over all possible values of *x*.

Equation (5) can be represented diagrammatically as in Figure 2. The probability of derived allele frequency *x* + *δx* at time *t* + *δt* is the product of the probability of moving from *p* to *x* in time *t* and the probability of moving from *x* to *x* + *δx* in time *δt*, summed over all possible values of *x*.

**Figure 3:**
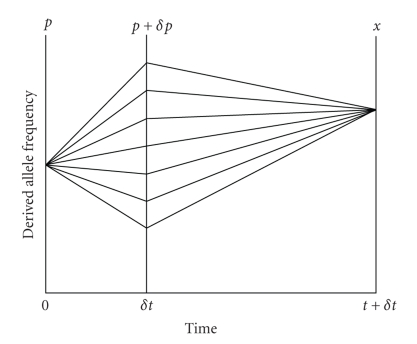
A diagrammatic intuition for (5) illustrates that the probability of frequency *x* at time *t* + *δt* is the product of the probability of moving from *p* to *p* + *δp* in time *δt* and the probability of moving from *p* + *δp* to *x* in time *t*, summed over all possible values of *δp*.

The frequency trajectory of a derived allele can also be depicted as in Figure 3, which illustrates that the probability of frequency *x* at time *t* + *δt* is the product of the probability of moving from *p* to *p* + *δp* in time *δt* and the probability of moving from *p* + *δp* to *x* in time *t*, summed over all possible values of **δ*p*. This is formalized as follows:

(8) $$f(x;\;p,t+\delta t)=\int_{0}^{1}f(p+\delta p;\;p,\delta t)f(x;\;p+\delta p,t)\;d(\delta p).$$

We can again perform a Taylor series expansion on both sides to derive the backward Kolmogorov equation:

(9) $$\frac{\partial f(x;p,t)}{\partial t}\;=\;b(p)\frac{\partial^{2}[f(x;p,t)]}{2\partial p^{2}}\;+a(p)\;\frac{\partial [f(x;p,t)]}{\partial p}.\;$$

**Figure 4:**
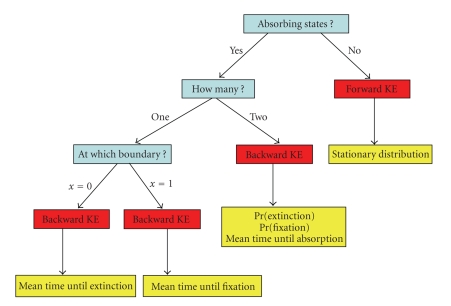
The utility of the Kolmogorov equations for studying evolutionary processes. Blue boxes correspond to questions that clarify the assumptions of the genetic model being used, the red boxes correspond to when the Kolmogorov equations (KEs) are utilized, and yellow boxes correspond to quantities of evolutionary interest.

The forward and backward Kolmogorov equations have played a central role in theoretical population genetics since 1922. For details regarding their derivation, we refer the reader [15, Chapter 4]. Next, we will discuss how they are utilized to derive formulas for various quantities of evolutionary interest (yellow boxes in Figure 4).

In a model where there is two-way recurrent mutation (i.e., there are no absorbing states, either extinction or fixation), stationarity is achieved when the probability of change in the derived allele frequency is no longer dependent on time *t*. We solve for the stationary distribution, *f(x)*, in the following manner. First, we integrate through the forward Kolmogorov equation with respect to *x*:

(10) $$\frac{\partial F(x;p,t)}{\partial t}\;=\;\frac{\partial \;[b(x)f(x;p,t)]}{2\partial x}\;-\;[a(x)f(x;p,t)],$$

(11) $$F(x;p,t)=\int_{0}^{x}f(y;p,t)\;dy,$$

where *F*(*x*; *p*, *t*) is the probability of the derived allele assuming a frequency between 0 and *x* at time *t*. Therefore, the derivative of *F*(*x*; *p*, *t*) with respect to *t* can be interpreted as the probability flux (change in probability over time) of the diffusion process. The stationary distribution, *f(x)*, can be solved by setting the probability flux equal to zero.

## 4. Derivation of Formulas Describing Evolutionary Processes of Interest

Let us now focus on a genetic model that assumes no recurrent mutation (i.e., two absorbing states, one at *x* = 0 and another at *x* = 1). As depicted by Figure 4, in such a model, it is possible to determine the probability of extinction (*x* = 0), the probability of fixation (*x* = 1), and the mean time until absorption (either at *x* = 0 or *x* = 1) by using the Kolmogorov backward equation (Figure 4). It is also possible to derive the mean time until absorption conditioned on always eventually reaching only one of the two states. Since this quantity is not directly applicable to the PRF, we do not review its derivation here, but instead refer the reader to [14].

### 4.1. Probability of Extinction

Using (11), we arrive at an equation parallel to (9):

(12) $$\frac{\partial F(x;p,t)}{\partial t}\;=\;b(p)\frac{\partial^{2}[F(x;p,t)]}{2\partial p^{2}}\;+a(p)\;\frac{\partial [F(x;p,t)]}{\partial p}.$$

The probability that the derived allele frequency, *x*, reaches 0 at or before time *t* follows from (11) and is given by

(13) $$P_{0}(p,t)=\int_{0}^{0^{+}}f(y;p,t)\;dy=F(0^{+};p,t),$$

where *p* is the initial frequency of the derived allele and 0^+^ indicates 0 + *ε*, where *ε* is very small.

Replacing *F*(0^+^; *p*, *t*) with *P*_0_(*p*, *t*), (12) can be written as

(14) $$\frac{\partial P_{0}(p,t)}{\partial t}\;=\;b(p)\frac{\partial^{2}[P_{0}(p,t)]}{2\partial p^{2}}\;+a(p)\;\frac{\partial [P_{0}(p,t)]}{\partial p}.$$

As *t* → ∞, *P*_0_(*p*, *t*) can be interpreted as the probability that extinction ever occurs (independent of time) and can be rewritten in the form *P*_0_(*p*). From (14), it is evident that *P*_0_(*p*) satisfies the following equation:

(15) $$0\;=\;b(p)\frac{\partial^{2}[P_{0}(p)]}{2\partial p^{2}}\;+a(p)\;\frac{\partial [P_{0}(p)]}{\partial p}.$$

Solving (15), we arrive at the following:

(16) $$P_{0}(p)=\frac{\int_{p}^{1}\psi (y)\;dy}{\int_{0}^{1}\psi (y)\;dy},$$

where

(17) $$\psi (y)=e^{-2\int^{y}[a(z)/b(z)]\;dz}$$

and where *a(z)* and *b(z)* are defined as in (6).

### 4.2. Probability of Fixation

The probability that the derived allele frequency, *x*, reaches 1 at time *t* follows from (11) and is given by

(18) $$\begin{array}{ll} P_{1}(p,t) & =\int_{1^{-}}^{1}f(y;p,t)\;dy\\ & =1-\int_{0}^{1^{-}}f(y;p,t)\;dy=1-F(1^{-};p,t), \end{array}$$

where *p* is the initial frequency of the derived allele and 1^−^ indicates 1 − *ε*, where *ε* is very small.

In (12), *F*(*x*; *p*, *t*) can be replaced by 1 − *F*(*x*; *p*, *t*) without any loss of generality. Also, by replacing 1 − *F*(1^−^; *p*, *t*) with *P*_1_(*p*, *t*), (12) can be rewritten as

(19) $$\frac{\partial P_{1}(p,t)}{\partial t}\;=\;b(p)\frac{\partial^{2}[P_{1}(p,t)]}{2\partial p^{2}}\;+a(p)\;\frac{\partial [P_{1}(p,t)]}{\partial p}.$$

By letting *t* → ∞ and solving for *P*_1_(*p*), we arrive at the following:

(20) $$P_{1}(p)=\frac{\int_{0}^{p}\psi (y)\;dy}{\int_{0}^{1}\psi (y)\;dy},$$

where *ψ*(*y*) has been defined in (17) and *a(z)* and *b(z)* have been defined in (6).

The probability of fixation and the probability of extinction must sum to 1. Using (16) and (20), we can verify that this is indeed the case.

Consider a genetic model that assumes the presence of selection, but no recurrent mutation, where *a*(*x*) = *sx*(1 − *x*) and *b*(*x*) = *x*(1 − *x*)/2*N*. Starting from (20), we can express the probability of fixation under this genetic model in the following manner:

(21) $$\begin{array}{ll} P_{1}(p) & =\frac{\int_{0}^{p}e^{-2\int^{y}\;[a(z)/b(z)]\;dz}dy}{\int_{0}^{1}e^{-2\int^{y}[a(z)/b(z)]\;dz}dy}\\ & =\frac{\int_{0}^{p}e^{-4Nsy}dy}{\int_{0}^{1}e^{-4Nsy}dy}=\frac{1-e^{-4Nsp}}{1-e^{-4Ns}}. \end{array}$$

### 4.3. Mean Time Until Either Extinction or Fixation

We define *ϕ*(*p*, *t*) to be the density function of the time *t* at which absorption occurs. The probability that absorption occurs, at either boundary *x* = 0 or *x* = 1, by time *t*, is

(22) $$P_{0}(p,t)+P_{1}(p,t)=\int_{0}^{t}\phi (p,t)\;dt.$$

Furthermore, since absorption must happen by *t* = ∞, we know that

(23) $$\int_{0}^{\infty }\phi (p,t)\;dt=1.$$

Performing integration by parts, we get the following:

(24) $$-1=-{\left[t\phi (p,t)\right]}_{0}^{\infty }+\int_{0}^{\infty }t\frac{\partial \phi (p,t)}{\partial t}\;dt.$$

Equations (14), (19), and (22) show that *ϕ*(*p*, *t*) satisfies the following equation:

(25) $$\frac{\partial \phi (p,t)}{\partial t}\;=\;b(p)\frac{\partial^{2}[\phi (p,t)]}{2\partial p^{2}}\;+a(p)\;\frac{\partial [\phi (p,t)]}{\partial p}.$$

Using (25) and the fact that *ϕ*(*p*, *t*) approaches 0 faster than *t* approaches ∞, we can rewrite ((24) as

(26) $$-1=0+\int_{0}^{\infty }t\;[b(p)\frac{\partial^{2}[\phi (p,t)]}{2^{*}\partial p^{2}}\;+a(p)\;\frac{\partial [\phi (p,t)]}{\partial p}]\;dt.$$

After interchanging the order of integration and differentiation we get

(27) $$-1=b(p)\frac{d^{2}\overset{¯}{t}(p)}{2^{*}dp^{2}}+a(p)\frac{d\overset{¯}{t}(p)}{dp},$$

where

(28) $$\begin{array}{ll} \overset{¯}{t}(p) & =\text{mean}\;\text{ time until absorption}\\ & =\int_{0}^{\infty }t\phi (p,t)\;dt=\int_{0}^{1}t(p,x)\;dx \end{array}$$

and *t*(*p*, *x*)*dx* is the mean time that the daf spends in the interval (*x*, *x* + *δx*) before absorption occurs.

We are interested in the case, where *p* = 1/2*N*, since this is the initial frequency of the derived allele. In this case, we are interested only in values of *x* greater than 1/2*N*, and for these values we can write

(29) $$t(p,x)=\frac{2P_{1}(p)\int_{x}^{1}\;\psi (y)\;dy}{b(x)\psi (x)},$$

and *ψ*(*x*) is defined in (17).

Under the simplest genetic model that assumes no selection and no recurrent mutation, we can set *s* = 0 in (17) and (21) and show that *P*_1_(*p*) reduces to *p* and *ψ*(*y*) reduces to 1. It follows from this that (29) can be reduced to

(30) $$t(p,x)=\frac{2p(1-x)}{x(1-x)/2N}=\frac{4Np}{x}.$$

Under a genetic model where *s* ≠ 0, using *γ* = 2*Ns*, (29) can be rewritten as

(31) $$t(p,x)=2N\frac{\left(2(1-e^{-2\gamma p})/\left(1-e^{-2\gamma }\right)\right)\;\int_{x}^{1}e^{-2\gamma y}\;dy}{x(1-x)(e^{-2\gamma x})}.$$

After integrating and simplifying the terms, we obtain

(32) $$t(p,x)=2N\frac{\left(1-e^{-2\gamma p})(1-e^{-2\gamma (1-x)}\right)}{\left[\gamma (1-e^{-2\gamma })]\;[x(1-x)\right]}.$$

Finally, substituting *γ* = 2*Ns* and *p* = 1/2*N*, and invoking the approximation *e* ^−*a*^ = (1 − *a*) for small values of *a*, *t*(*p*, *x*) reduces approximately to

(33) $$f(x)\;=t(p,x)\approx \;\frac{2(1-e^{-2\gamma (1-x)})}{\left[(1-e^{-2\gamma })]\;[x(1-x)\right]}\;,$$

where *f*(*x*)*dx* is a notation common in the literature to represent the expected time for which the population frequency of a derived allele is in the range (*x*, *x* + *dx*) before eventual absorption.

## 5. Poisson Random Field Theory

S. Sawyer and D. Hartl expanded the modeling of site evolution to multiple sites. Their model makes the following assumptions: (1) mutations arise at Poisson times, (2) each mutation occurs at a new site (infinite sites, irreversible), and (3) each mutant follows an independent WF process (no linkage). Sawyer and Hartl noticed from *f*(*x*) in (33), that

(34) $$\int_{x_{1}}^{x_{2}}\theta \;f(x)\;dx=\int_{x_{1}}^{x_{2}}g(x)\;dx$$

is the expected number of sites in the population with derived allele frequency between *x_1_* and *x*_2_ (where *θ* equals 2*Nμ*, the per-locus mutation rate). The function *g*(*x*), for which the full expression is given below, is also referred to in the literature as the limiting, equilibrium, or expected density function for derived allele frequencies.

(35) $$g(x)=\theta \;\frac{2(1-e^{-2\gamma (1-x)})}{\left[(1-e^{-2\gamma })]\;[x(1-x)\right]}=4N\mu \frac{1-e^{-2\gamma (1-x)}}{\left(1-e^{-2\gamma })\;x(1-x\right)}.$$

In a sample of size *n*, the expected number of sites with *i* (which ranges from 1 to *n* − 1) copies of the derived allele is defined as a function of *g*(*x*):

(36) $$F(i)\;=\int_{0}^{1}\;g(x)\;P(i\;|\;x)\;dx\;=\;\int_{0}^{1}\;g(x)\;\left(\begin{array}{c} n\\ i \end{array}\right)\;x^{i}\;{\left(1-x\right)}^{n-i}\;dx.$$

The intuition behind *F(i)* is the following. The expected number of polymorphic sites with population daf *x* that have *i* copies of the derived allele out of *n* samples is given by the product of the expected number of sites with population daf *x*, *g*(*x*), and the probability that each of those sites has *i* copies in the sample, which is given by the binomial calculation in the right-hand side of (36). To determine the expected number of sites with *any* population daf that have *i* copies of the derived allele, this product must be integrated over all possible values of *x* (resulting in *F(i)* above).

Consider the sample data *X* = (*X*_1_, *X*_2_, *X*_3_,…, *X*_*n*−1_), where *X*_*i*_ is the observed number of sites with *i* copies of the derived allele out of *n*. Sawyer and Hartl showed that the number of derived alleles in the entire population at a particular frequency is a PRF with mean density given by (35) [2]. It follows, from the marking theorem on Poisson processes [16], that each random variable *X_i_* is an independent Poisson distribution with mean equal to *F(i)* [2]. This framework allows us to define the probability of observing *x_i_* sites that have *i* copies of the derived allele (and *n* − *i* copies of the ancestral allele) as the following:

(37) $$P(X_{i}=x_{i}\;|\;\theta ,\gamma )\;=\;\frac{e^{-F(i)}F{\left(i\right)}^{x_{i}}}{x_{i}!}.$$

Since the *X_i_*'s are independent, the probability of observing *X* = (*X*_1_, *X*_2_, *X*_3_,…, *X*_*n*−1_) is given as

(38) $$P(X)\;=\;L(\theta ,\;\gamma )\;=\;\overset{n-1}{\underset{i=1}{\prod }}P(X_{i}=x_{i}\;|\;\theta ,\gamma ).$$

The likelihood equation above provides a convenient means of estimating the values of the parameters **θ** and **γ**. The use of the PRF theory leads directly to a likelihood-ratio test of neutrality. *Λ* is defined as the ratio of the likelihood value under the maximum likelihood estimate of **γ** to the likelihood value under the neutral value of **γ**. It is a standard result that 2lnΛ is asymptotically chi-square distributed with one degree of freedom [17].

Sawyer and Hartl further extended the PRF model in order to calculate the ratio of expected number of polymorphisms within species to expected number of fixed differences between species.In 1991, McDonald and Kreitman devised a 2-by-2 contingency table test of neutrality that was later named the MK test [18]. In the traditional MK test, a 2-by-2 contingency table is formed in order to compare the number of nonsynonymous and synonymous sites that are polymorphic within a species (RP and SP) and diverged between species (RF and SF) (Table 1). The central assumption of the MK test is that only nonsynonymous sites may be under selective pressure (i.e., synonymous sites are assumed to be neutrally evolving). If nonsynonymous sites are evolving according to a neutral model, then the expectation is that *P*_*n*_/*P*_*s*_ = *D*_*n*_/*D*_*s*_. However, if nonsynonymous sites are under negative selection, then the expectation is that *P*_*n*_/*P*_*s*_ > *D*_*n*_/*D*_*s*_, and if under positive selection, then *P*_*n*_/*P*_*s*_ < *D*_*n*_/*D*_*s*_. Sawyer and Hartl derived the formulas for the expected values of SP, SF, RP, and RF using their PRF theory [2]. Below are the derivations of each of these formulas. For all of the derivations, assume that the data consists of samples of size *m* and *n* from two different species.

### 5.1. Expected Number of Synonymous Polymorphic Sites

Under neutral evolution (*s* = 0), the expected number of polymorphic sites with population daf *x* can be computed by taking the product of the per-locus mutation rate (*θ* = 2*Nμ*) and the probability under a neutral model of a single mutation having a frequency of *x* (from (30)):

(39) $$g_{\text{neutral}}(x)=\;\theta \frac{4Np}{x}=2N\mu \frac{4N(1/2N)}{x}=\frac{4N\mu }{x}=\frac{2\theta }{x}.$$

Now, consider species 1 with sample size *m*. The probability that a polymorphic site, with population daf equal to *x*, is detected as polymorphic in a sample of size *m* is given as

(40) $$\begin{array}{ll} P_{m}(x) & \;=\;1-(\text{all}\;\;m\text{ are derived})-(\text{all}\;\;m\text{ are}\;\text{ ancestral})\\ & \;=\;1-x^{m}-{\left(1-x\right)}^{m}. \end{array}$$

The expected number of synonymous polymorphic sites, with population daf *x*, in the species 1 sample is the product of the expected number of synonymous polymorphic sites with daf *x* in the population (*g*_neutral_(*x*)) and the fraction of those that are expected to be detected in a sample of size *m*(*P*_*m*_(*x*)). It follows then that the total expected number of synonymous polymorphic sites, with any population daf, in the species 1 sample is computed by integrating the product of *g*_neutral_(*x*) and *P*_*m*_(*x*) over the range of possible values for *x*:

(41) $$\begin{array}{ll} L(m) & \;=\int_{0}^{1}g_{\text{neutral}}(x)\;P_{m}(x)\;dx\\ & =2\theta \;\int_{0}^{1}\frac{1-x^{m}-{\left(1-x\right)}^{m}}{x}\;dx\\ & =2\theta \overset{m-1}{\underset{k=1}{\sum }}\frac{1}{k}. \end{array}$$

Finally, the total number of expected synonymous polymorphic sites in both species' sample data is given as

(42) SP=L(m)+L(n).

### 5.2. Expected Number of Replacement Polymorphic Sites

The derivation of the expected value of RP follows the same logic. As described in (35), the expected number of polymorphic sites with population daf *x* given some average selection pressure *γ* is given by *g(x)*. Similar to (41), the total expected number of replacement polymorphic sites in the species 1 sample is computed by integrating the product of *g(x)* and *P_m_(x)* from 0 to 1:

(43) $$\begin{array}{ll} H(m) & =\int_{0}^{1}\;g(x)\;P_{m}(x)dx\\ & =\int_{0}^{1}g(x)\;[1-x^{m}-{\left(1-x\right)}^{m}]\;dx. \end{array}$$

Finally, the total expected number of replacement polymorphic sites in both species' sample data is given as

(44) RP=H(m)+H(n).

### 5.3. Expected Number of Synonymous Fixed Substitutions

When *s* = 0, the expected number of fixed substitutions in one species relative to another that diverged *t*_div_2*N* generations ago is given as the product of the number of total mutations and the probability of fixation of each mutation. The number of total mutations is the product of the mutation rate per generation and the number of generations since divergence is

(45) $$\theta t_{\text{div}}2N.$$

The probability of fixation is given in (21). As *s* approaches 0 (i.e., neutral evolution), the probability of fixationcan be reduced to *p* using the approximation *e* ^−*a*^ = (1 − *a*) for small values of *a*. Thus, for a newly derived neutral allele that has an initial frequency of 1/2*N*, the probability of fixation is also 1/2*N*.

Therefore, the total expected number of fixed substitutions in species 1 is

(46) $$(\theta t_{\text{div}}2N)(\frac{1}{2N})=\theta t_{\text{div}}.$$

However, given that the data are samples of the populations from both species, not all sites identified as fixed substitutions in the sample are truly fixed substitutions in the entire population. The expected number of sites in the species 1 sample that fall into this category is given by

(47) $$\int_{0}^{1}T_{m}(x)\;g_{\text{neutral}}(x)\;dx=\theta \;\int_{0}^{1}\left(x^{m}\;\frac{2}{x}\;\right)\;dx=\theta \;{\left|\frac{x^{m}}{m}\right|}_{0}^{1}=\theta \;\frac{2}{m},$$

where *T*_*m*_(*x*) = Pr(a derived allele daf *x* < 1 is observed with *x* = 1 in a size *m* sample) and *g*_neutral_(*x*) is given in (39).

Therefore, the total expected number of synonymous fixed substitutions in both species' sample data is given as

(48) $$\text{SF}=\theta (t_{\text{div}}+\frac{2}{m})+\theta (t_{\text{div}}+\frac{2}{n})=2\theta (t_{\text{div}}+\frac{1}{m}+\frac{1}{n}).$$

### 5.4. Expected Number of Replacement Fixed Substitutions

Similar to the calculation of (46), given some selection pressure, **γ**, the expected number of fixed substitutions in one species relative to another that diverged *t*_div_2*N* generations ago is given as the product of (45) and (21):

(49) $$(\theta \;t_{\text{div}}2N)\;(\frac{1-e^{-4Nsp}}{1-e^{-4Ns}}).$$

Substituting 1/2*N* for *p* and invoking the approximation that *e* ^−*a*^ = (1 − *a*) for small values of *a*, we arrive at the following:

(50) $$(\theta \;t_{\text{div}}2N)\;(\frac{2s}{1-e^{-2\gamma }})=\theta \;t_{\text{div}}\frac{2\gamma }{1-e^{-2\gamma }}.$$

However, again, given that the data are samples of the populations from both species, not all sites identified as fixed substitutions in the sample are truly fixed substitutions in the entire population. The expected number of sites in the species 1 sample that fall into this category is given by

(51) $$Q(m)=\;\int_{0}^{1}T_{m}(x)\;g(x)\;dx=2\theta \;\int_{0}^{1}x^{m-1}\frac{1-e^{-2\gamma (1-x)}}{\left(1-e^{-2\gamma })(1-x\right)}\;dx.$$

Therefore, the total expected number of replacement fixed substitutions in both species' sample data is given as

(52) $$\begin{array}{ll} \text{RF} & =\theta \;(\frac{2\gamma \;t_{\text{div}}}{1-e^{-2\gamma }}+2G(m))+\theta \;(\frac{2\gamma \;t_{\text{div}}}{1-e^{-2\gamma }}+2G(n))\\ & =2\theta \;(\frac{2\gamma \;t_{div}}{1-e^{-2\gamma }}+G(m)+G(n)),\\ & \;\text{where }G(m)=Q(m)/2\theta . \end{array}$$

### 5.5. Estimating Parameters

It is possible to obtain estimates of **θ** and **γ** by setting each of the observed values SP, RP, SF, and RF (Table 1) to their PRF expectations given by (42), (44), (48), and (52), respectively, and solving for the parameters. It has been shown that these estimates are equivalent to maximum-likelihood estimates [2, 19]. Bustamante et al. also eloquently describe and implement a hierarchical Bayesian model for parameter estimation [9].

## 6. Concluding Remarks

Sawyer and Hartl's seminal presentation of the PRF in 1992 provided an innovative mathematical framework for estimating selection pressures and mutation rates, which are critical parameters that influence molecular evolution. However, it is worth noting that the model does harbor certain limitations. Foremost among these is the assumption of site independence, which is equivalent to the assumption of free recombination among mutations (i.e., no linkage). Thus, the model may not be appropriate for many data wherein strong linkage is present. Another limitation is the assumption of infinite sites (i.e., each mutation is at a new site). Although this assumption allows for a simpler model, it is not always biologically appropriate, especially for organisms that experience a higher mutation rate. Indeed, recent work has shown that the assumption of infinite sites can underestimate selection pressures and mutation rates and even infer positive selection, when in fact there is weak negative selection [20]. Recent theoretical work has focused on relaxing these and other assumptions of the original PRF model, so as to make it more appropriate for diverse biological contexts. For a brief list of such studies, we refer the reader to [20]. Ongoing theoretical and empirical work in this area will undoubtedly continue to extend the power of a PRF-based approach for population genetic inference.

## Figures and Tables

**Table 1:** *McDonald-Kreitman contingency table.* 2-by-2 contingency table introduced by [18] for the inference of natural selection on nonsynonymous coding sites.

MK Table	No. of polymorphic sites	No. of fixed substitutions
Synonymous	SP	SF
Replacement (nonSynonymous)	RP	RF
